# Supplementary material for: High-Throughput Screening Identified Compounds Sensitizing Tumor Cells to Glucose Starvation in Culture and VEGF Inhibitors In Vivo
Source: Cancers (Basel). 2019 Jan 30;11(2):156. doi: 10.3390/cancers11020156 (PMC6406438; doi:10.3390/cancers11020156)
Supplement: Supplementary file 1 [file cancers-11-00156-s001.pdf]

High-Throughput Screening Identified Compounds Sensitizing Tumor Cells to Glucose Starvation in Culture and VEGF Inhibitors in Vivo

Ran Marciano, Manu Prasad, Tal Ievy, Sapir Tzadok, Gabriel Leprivier, Moshe Elkabets, Barak Rotblat

Supplementary Material

Table S1. List of compounds identified in the HTP.

Molecule Name	Compound Name	SecName	IC50 Glucose Depletion: IC50 (µM)	IC50 Glucose Rich: IC50 (µM)	Reference
PCM-0095674	QNZ		< 0.2	14	1
PCM-0095313	GSK1292263		4.1	> 15.0	
PCM-0095252			< 0.2	> 15.0	
PCM-0094997	Amuvatinib (MP-470)		0.9	> 15.0	
PCM-0091403		DICHLOROEVERNIC ACID	2.3	> 15.0	
PCM-0091267		alpha-TOXICAROL (dl)	< 0.2	> 15.0	2
PCM-0091223		beta-TOXICAROL	< 0.2	> 15.0	2
PCM-0091193		MUNDULONE ACETATE	1.2	12	
PCM-0091161		ANTIMYCIN A	< 0.2	> 15.0	3
PCM-0091132		DEOXYSAPPANONE B TRIMETHYL ETHER	4.4	> 15.0	
PCM-0091067		MUNDULONE	0.4	5	2
PCM-0091044		IRIGINOL HEXAACETATE	2.4	> 15.0	
PCM-0090999		DEGUELIN(-)	< 0.2	10.7	4
PCM-0090975		3-METHOXYCATECHOL	2.3	7.1	5
PCM-0090931		2,3,4-TRIHYDROXYBENZALDEHYDE	2.3	> 15.0	
PCM-0090898		MUNDOSERONE	0.6	> 15.0	2
PCM-0090870		ISOROTENONE	< 0.2	> 15.0	2
PCM-0090710		CHLORMIDAZOLE	0.7	> 15.0	
PCM-0090649		BERBERINE CHLORIDE	4.5	> 15.0	6
PCM-0090425	CONCEPTROL	NONOXYNOL-9	0.7	> 15.0	
PCM-0090329	ROCCAL	BENZALKONIUM CHLORIDE	1.6	5.3	
PCM-0090062	CEPACOL	CETYLPYRIDINIUM CHLORIDE	0.5	5	

PCM-0086543	Benzethonium chloride		0.8	6.8	7
PCM-0086538	Methyl benzethonium chloride		2	9.4	7
PCM-0085933	Clofilium tosylate		4	13.8	
PCM-0085900	Antimycin A		< 0.2	> 15.0	8
PCM-0065499	MG 624	N,N,N-Triethyl-2-(4-trans-stilbenoxy)ethylammonium iodide	4.6	> 15.0	
PCM-0065075	Dequalinium dichloride		2.1	14.8	9
PCM-0065000	Papaverine hydrochloride		3.6	> 15.0	10
PCM-0064878	Pentamidine isethionate	4'4-[1,5-Pentanediylbis(oxy)]bis-benzenecarboximidamide isethionate	4.9	> 15.0	11
PCM-0064697	Benserazide		4.6	> 15.0	

Table S2. Validated compounds found to selectively reduce cell viability upon glucose starvation.

	Viability normailzed to control		
	Glucose free media	Complete media	delta
PCM-0085900-001	−89.3	21.6	110.9
PCM-0090931-001	−93.2	8.8	102.0
PCM-0091044-001	−96.3	5.4	101.6
PCM-0091403-001	−93.0	−4.4	88.6
PCM-0090975-001	−87.1	−5.5	81.6
PCM-0090999-001	−83.1	−1.7	81.4
PCM-0091161-001	−87.3	−10.7	76.6
PCM-0086538-002	−79.3	−3.5	75.8
PCM-0091067-001	−98.3	−24.1	74.1
PCM-0091193-001	−74.6	−4.7	69.8
PCM-0064697-003	−55.8	13.0	68.9
PCM-0091223-001	−77.8	−9.2	68.6
PCM-0091267-001	−81.1	−13.2	67.9
PCM-0090870-001	−83.5	−15.8	67.7
PCM-0090425-001	−85.6	−18.7	66.9
PCM-0086538-001	−76.7	−10.2	66.5
PCM-0095330-001	−77.74939	−11.9	65.8
PCM-0090898-001	−75.7	−12.4	63.3

PCM-0065189-002	−50.9	5.2	56.1
PCM-0090710-001	−79.6	−23.9	55.7
PCM-0091069-001	−57.3	−2.6	54.7
PCM-0064624-001	−51.4	3.0	54.4
PCM-0094951-001	−64.47413	−10.2	54.3
PCM-0086543-002	−84.5	−34.9	49.7
PCM-0086543-001	−84.8	−36.1	48.7
PCM-0085888-001	−81.0	−33.0	48.1
PCM-0065075-003	−51.2	−3.2	48.0
PCM-0065075-002	−62.2	−14.7	47.5
PCM-0094992-001	−55.68146	−8.2	47.5
PCM-0090329-001	−74.1	−29.4	44.7
PCM-0090062-001	−90.2	−46.5	43.7
PCM-0065309-001	−80.5	−37.3	43.2
PCM-0065564-001	−98.7	−56.4	42.3
PCM-0086075-002	−52.7	−11.0	41.7
PCM-0086501-002	−55.6	−14.0	41.5
PCM-0064957-002	−66.4	−25.3	41.1
PCM-0064697-002	−52.2	−11.3	40.9
PCM-0094958-001	−58.10148	−18	40.1
PCM-0090990-001	−82.5	−42.9	39.6
PCM-0085933-001	−55.5	−16.4	39.1
PCM-0065000-002	−50.0	−11.6	38.5
PCM-0065022-002	−52.92857	−15.6	37.3
PCM-0064420-001	−95.0	−57.8	37.2
PCM-0085936-001	−98.6	−61.9	36.7
PCM-0095063-001	−81.88943	−47.22	34.7
PCM-0091204-001	−54.9	−20.4	34.4
PCM-0065382-001	−70.0	−36.0	34.0
PCM-0065190-001	−86.3	−53.3	33.0

PCM-0090700-001	−92.5	−59.8	32.7
PCM-0091352-001	−98.0	−65.3	32.7
PCM-0095675-001	−72.9365	−41.4	31.5
PCM-0064596-001	−51.0	−20.4	30.5
PCM-0065075-001	−75.1	−45.1	30.0
PCM-0091392-001	−61.6	−31.7	29.9
PCM-0095398-001	−72.75871	−44.6	28.2
PCM-0086151-001	−64.0	−37.4	26.7
PCM-0086012-002	−55.22988	−29	26.2
PCM-0095292-001	−72.70541	−47.1	25.6
PCM-0064895-005	−66.01478	−40.7	25.3
PCM-0090803-001	−66.2	−43.1	23.1
PCM-0090712-001	−61.3	−38.5	22.8
PCM-0090714-001	−50.2	−27.9	22.4
PCM-0095144-001	−61.3049	−39.6	21.7
PCM-0095710-001	−58.53656	−37.2	21.3
PCM-0086254-001	−57.9	−40.4	17.5
PCM-0064890-002	−62.73515	−47.5	15.2
PCM-0094965-001	−54.38876	−48.5	5.9

References

1. Krishnathas, R.; Bonke, E.; Dröse, S.; Zickermann, V.; Nasiri, H.R. Identification of 4-N-[2-(4-phenoxyphenyl)ethyl]quinazoline-4,6-diamine as a novel, highly potent and specific inhibitor of mitochondrial complex I. *Medchemcomm* **2017**, *8*, 657–661.
2. Gohil, V.M.; Sheth, S.A.; Nilsson, R.; Wojtovich, A.P.; Lee, J.H.; Perocchi, F.; Chen, W.; Clish, C.B.; Ayata, C.; Brookes, P.S.; Mootha, V.K. Nutrient-sensitized screening for drugs that shift energy metabolism from mitochondrial respiration to glycolysis. *Nat. Biotechnol.* **2010**, *28*, 249–255.
3. Lipton, S.H. Factors Affecting the Mitochondrial the Binding Resrkatorv of Antimycin Chain. *Biol. Chem.* **1967**, *242*, 4888–4896.
4. Hail, N. Lotan, R. Apoptosis induction by the natural product cancer chemopreventive agent deguelin is mediated through the inhibition of mitochondrial bioenergetics. *Apoptosis* **2004**, *9*, 437–447.
5. Cheng, S.C.; Pardini, R.S.; Lee, A.M. Structure–Inhibition relationships of various phenolic compounds towards mitochondrial respiration. *Pharmacol. Res. Commun.* **1978**, *10*, 897–910.
6. Mikeš. V.; Yaguzhinskij, L.S. Interaction of fluorescent berberine alkyl derivatives with respiratory chain of rat liver mitochondria. *J. Bioenerg. Biomembr.* **1985**, *17*, 23–32.

7. Yip, K.W.; Mao, X.; Au, P.Y.B.; Hedley, D.W.; Chow, S.; Dalili, S.; Mocanu, J.D.; Bastianutto, C.; Schimmer, A.; Liu, F.F. Benzethonium chloride: A novel anticancer agent identified by using a cell-based small-molecule screen. *Clin. Cancer Res.* **2006**, *12*, 5557–5569.
8. Kim, H.; Esser, L.; Hossain, M.B.; Xia, D.; Yu, C.-A.; Rizo, J.; van der Helm, D.; Deisenhofer, J. Structure of Antimycin A1, a Specific Electron Transfer Inhibitor of Ubiquinol−Cytochrome c Oxidoreductase. **1999**, *121*, 4902–4903.
9. Anderson, W.M.; Patheja, H.S.; Delinck, D.L; Baldwin, W.W; Smiley, S.T.; Chen, L.B. Inhibition of bovine heart mitochondrial and Paracoccus denitrificans NADH----ubiquinone reductase by dequalinium chloride and three structurally related quinolinium compounds. *Biochem. Int.* **1989**, *19*, 673–685.
10. Santi, R.; Ferrari, M.; Contessa, A.R. On the mechanism of spasmolytic effect of papaverine and certain derivatives. *Biochem Pharmacol* **1964**, *13*, 153–158.
11. Moreno, S.N.J. Pentamidine is an uncoupler of oxidative phosphorylation in rat liver mitochondria. *Arch. Biochem. Biophys.* **1996**, *326*, 15–20.
